# Responsiveness of the Short Warwick Edinburgh Mental Well-Being Scale (SWEMWBS): evaluation a clinical sample

**DOI:** 10.1186/s12955-018-1060-2

**Published:** 2018-12-22

**Authors:** Neha Shah, Mizaya Cader, William P. Andrews, Dilini Wijesekera, Sarah L. Stewart-Brown

**Affiliations:** 1 Public Health Registrar, Warwick Medical School University of Warwick, Coventry, UK; 2 Consultant Community Physician, Ministry of Health, Colombo, Sri Lanka; 3 Independent Researcher/ Consultant at Pragmatic Research Network, Rhosneigr, UK; 4 Professor of Public Health, Warwick Medical School University of Warwick, Coventry, UK

## Abstract

**Background:**

SWEMWBS is a popular measure of mental wellbeing, shown to be valid in clinical populations. Responsiveness to change has not yet been formally assessed.

**Methods:**

Analysis of data from a clinical sample of 172 clients undergoing up to 4 sessions of cognitive hypnotherapy. Cohen’s D effect size (ES), Standardised response mean (SRM), probability of change statistic (P^) were used to evaluate whether SWEMWBS detected statistically important changes at the group level. Cohen’s D effect size (ES) and Standard error of measurement (SEM) and were used to evaluate whether SWEMWBS detected statistically important changes at the individual level.

**Results:**

Mean (SD) SWEMWBS scores increased from baseline to therapy 4 from 19.28 (3.921) to 23.32 (4.873). At group level, using Cohen’s D effect size, improvement ranges from ES = 0.20–1.41 and using SRM, ranged from 0.30–0.88, increasing with number of therapy sessions. (P^) ranged from 0.65–0.8. At individual level, use of Cohens D ES > 0.5 indicated statistically important improvement in 29.9–86.1% cf. 20.1–80.6% using a standard of 2.77 SEM (2.87 points). The lower threshold of 1 SEM (1.03 points) indicated statistically important improvement in 43.0–81.0%.

**Conclusion:**

SWEMWBS is responsive to change at individual and group level. At individual level a change of between 1 and 3 points meets thresholds for statisticially important change, depending on standard used. Anchor based studies are necessary to confirm that such change represents minimally important change from the perspective of study participants.

## Background

Mental wellbeing, the positive aspect of mental health, is a core concept for public mental health and mental health promotion [1]. The Warwick-Edinburgh Mental Well-Being Scale (WEMWBS) was developed in 2007 to support public mental health by enabling the monitoring of mental wellbeing, investigation of determinants and evaluation of interventions [2]. The conceptual framework behind WEMWBS reflects growing consensus that mental wellbeing consists of two key dimensions: feeling good or hedonia, and functioning well or eudaimonia [3, 4]. A seven item version, the Short Warwick–Edinburgh Mental Well-Being Scale (SWEMWBS) was resolved in 2009 using Rasch modelling. This offers superior interval scaling to WEMWBS [5]. Robust measurement properties combined with brevity make SWEMWBS popular for monitoring mental wellbeing in populations.

Mental wellbeing is now also beginning to be recognised as an outcome of importance in mental health services [6] where valid outcome measures, usually based on patient self-report, are required by commissioners to monitor the effectiveness of service provision [7]. These measures may also be valuable to clinicians for monitoring treatment progress and aiding clinical decision-making [8]. Research has suggested that WEMWBS and SWEMWBS are well liked by service users and carers, who value the positive wording, self-administered nature and appropriate length [6]. Whilst WEMWBS responsiveness to change has been demonstrated [9], responsiveness of SWEMWBS to change in clinical and community settings has not been formally evaluated.

Responsiveness covers an instrument’s ability to accurately detect meaningful change when change occurs. Minimal important change can be defined as minimum change which is of significance to the patient, member of the public or the health professional, that exceeds variation attributable to chance [10, 11–13]. There are two broad methods for measuring responsiveness: distribution based, where the observed change is compared to the statistical properties of the sample, which measures variation attributable to chance; and instrument or anchor based, where the observed change is related to an external criterion of change and in clinical populations measures clinical significance [14]. Meaningful change can be different at group and individual level.

The aim of this study was to evaluate the responsiveness of SWEMWBS using distributional methods in a clinical sample of cognitive hypnotherapy service users at group and individual level, and thus provide further evidence regarding its’ suitability as an outcome measure in clinical practice.

## Methods

Data for this study were collected by cognitive hypnotherapists at Quest Cognitive Hypnotherapy (QCH) during routine clinical practice using the Pragmatic Research Network’s electronic software (Pragmatic Tracker) [15]. The latter is a collaboration of professionals promoting service-based evaluation and feedback-informed treatment which developed Pragmatic Tracker to allow session by session administration of outcome measures, with feedback to clients, therapists and service evaluators.

Therapists were informed about the proposed study through the Quest online forum by the project coordinator and were invited to participate in the research project. All therapists worked in private practice with fee-paying clients in a range of locations throughout the UK. The research project was overseen by the pragmatic research network providing an initial training day and combination of face-to-face, telephone and e-mail support.

### Participants

Participants were adult clients seeking cognitive hypnotherapy (CHT) as treatment for mental health problems, mainly anxiety and depression, at the QCH practices of the participating therapists between October 2014 and April 2016. 167 participants were recruited at initial session, 36 of whom provided data for 4 sessions.

### Intervention

CHT is a type of therapy which uses induction of the patient into a trance like state to access unconscious problematic thoughts, feelings and memory patterns. At the initial assessment, the therapist identifies the client’s use of language and the unconscious phenomena they experience while acting within their problem pattern. Subsequent sessions build on these findings focusing on interrupting faulty pattern matching by changing the context, structure, process or consequence (the four quadrants) of the problem pattern. Each technique or intervention acts within a specific quadrant, so treatment is highly individualised, based on the content of the client’s unique problem pattern [15]. There is no set amount of treatment sessions; length and frequency of treatment is negotiated between therapist and client based upon progress and ongoing need.

### Outcome measure

SWEMWBS was selected as one of several outcome measures for inclusion in the pragmatic tracker software because therapists found it helpful to monitor treatment from a positive perspective. Because intervals between sessions are often one week long, SWEMWBS was presented with a one rather than two week time frame for response options All participants were informed about QCH research objectives and written consent for outcome monitoring was obtained at the first session. Individual client data were gathered before assessment and at each subsequent session by self-administration using web based ‘pragmatic tracker’ software, either remotely via email link or on arrival at the clinic.

### Statistical analysis

Analyses were performed using the SPSS (v23.0, IBM) and MedCalc (version 17.9, MedCalc Software) packages. Normality of distribution for SWEMWBS across participants was assessed by visual inspection of the histogram and using the Shapiro-Wilk test for normality. Descriptive statistics including the mean and standard deviation (SD) of SWEMWBS score at each session were calculated.

There is no clear consensus regarding which statistical standards should be used to assess responsiveness. We used four distributional methods [11, 13, 14, 16]: Cohen’s D effect size and Standardized Response Mean (SRM) for group level analysis [9, 11, 17] and Cohen’s D effect size and Standard Error of the Mean (SEM) for individual level analysis [10, 18].

### Group level analysis

Distribution of scores was investigated using a paired t test for group level analysis between assessment and each time point up to 4 therapy sessions. Cohen’s D was calculated by dividing the mean difference of paired measurements between assessment and each time point by the pooled Standard Deviation (SD) of assessment and the respective time point. The standard cut off values for Cohen’s D; ‘trivial’ (ES < 0.20), ‘small’ (ES ≥ 0.20 < 0.50), ‘moderate’ (ES ≥ 0.50 < 0.80), or large (ES ≥ 0.80) were used to describe statistically meaningful change at group level [17].

SRM was calculated by dividing the mean difference of paired measurements between assessment and each time point by the standard deviation of the differences between the paired measurements [11]. SRM was interpreted by calculating the probability of change statistic P, which relates to the cumulative normal distribution function of the derived SRM. The P statistic denotes the probability that the instrument detects a change, intuitively representing the proportion of subjects whose scores have changed, and ranges from 0.5 (no ability to detect change) to 1 (perfect ability to detect change) [19]. The 95% CI of the P statistic was estimated using the substitution method, which uses the cumulative standard normal distribution function of the SRM to calculate the respective lower and upper limit [20]. 95% confidence intervals for the Cohen’s D and SRM were calculated using bootstrapping with 1000 different combinations from the existing data to derive lower and upper limit.

### Individual level analysis

Cohen’s D was calculated for every individual by dividing the difference between assessment score and score at each therapy session up to 4 sessions by the pooled SD of assessment and respective session score. A threshold of ES > 0.5 was chosen as a cut off for statistically meaningful change at individual level, as recommended by Norman et al. after a review of various distributional and anchor based methods for establishing minimal important difference [18]. The proportion of patients with improvement after each therapy session was calculated.

SEM of the instrument was calculated as; SEM = SD (baseline) *√1-rxx (internal consistency reliability of the instrument). Cronbach’s alpha was used to calculate the reliability statistic. Different thresholds ranging from 1 SEM to 2.77 SEM have been proposed to consider individual level change as statistically meaningful [21]. As the SEM of a measure is said to be independent of the sample [21], a single value change value can be applied to denote improvement across different samples. A threshold of 2.77 was chosen which takes into account measurement error, the combined variability across baseline and post intervention samples, and chance at the 95% confidence interval [22]. The proportion of individuals with change score more than 2.77 SEM was calculated for each therapy session to determine the proportion with statistically meaningful improvement. Given discrepancy between the two approaches, kappa statistics were used to analyse method agreement [23]. For comparison levels of statistically meaningful change were also calculated using thresholds of 1 SEM and 2 SEM.

## Results

The mean age of the participants was 40.6 years (*N* = 172; SD = 12.71). The majority, 74.4% (*n* = 128) were female, white British (73.8%, *N* = 127) and employed (75.6%, *N* = 130). 167 participants completed SWEMWBS at assessment; of these 134 of completed data before the first therapy session, 95 before the second, 66 the third and 36 the fourth. There was a mean duration of 12 days between assessment and session 1, 13 days between sessions 1 and 2, 21 days between sessions 2 and 3, and 22 days between sessions 3 and 4. The mean value of SWEMWBS at the assessment session was 19.28 (SD = 3.921). No significant difference in baseline SWEMWBS was found between groups of clients that attended one, two three and four sessions respectively. Scores increased linearly at each therapy session to reach a mean value of 23.32 (SD = 4.873) before therapy session 4 (see Table 1). Normality of distribution was confirmed on visual inspection and using Shapiro-Wilk testing.

**Table 1:** Distribution of mean SWEMWBS score at assessment and after therapy session

	N	Mean	SD	95% CI mean	Min	Max
SWEMWBS at Assessment	167	19.28	3.921	18.66–19.89	7	35
SWEMWBS before Therapy 1	134	20.70	4.360	19.97–21.38	7	35
SWEMWBS before Therapy 2	95	21.60	5.343	20.57–22.73	7	35
SWEMWBS before Therapy 3	66	22.47	4.383	21.44–23.48	12.4	35
SWEMWBS before Therapy 4	36	23.32	4.873	21.77–24.85	9.51	35

Group level analysis is shown in Table 2. Using Cohen’s D a small change of SWEMWBS was observed from assessment to subsequent therapy session 1 (ES = 0.33; 95%CI 0.20–0.46), a moderate change from assessment to therapy session 2 (ES =0.67; 95%CI 0.48–0.86) and large changes from assessment to therapy 3 (ES = 0.92 95%CI 0.69–1.15) and therapy 4 (ES = 1.03; 95% CI 0.60–1.41). The SRM ranged from 0.49 (95% CI 0.30–0.65) to 1.01 (95% CI 0.63–1.36) and the probability of change statistic P from 0.69 (95% CI; 0.65–0.72) to 0.84 (95% CI; 0.80–0.88). The lower 95% CI of probability of detecting a statistically meaningful change was 0.65 from assessment to therapy 1, 0.71 from assessment to therapy 2, 0.77 from assessment to therapy 3 and 0.80 from assessment to therapy 4.

**Table 2:** Evaluation of responsiveness of SWEMWBS at group level

Mean and SD				Indices of Responsiveness					
				Cohen’s D		SRM			
Time points	Paired mean difference	Pooled SD	SD of paired difference	Effect Size (Cohen’d D)	95% CI of Cohen’s D	SRM	95% CI of SRM	Probability (P^)	95% CI of probability
Assessment – Therapy 1 (*N* = 134)	1.38	4.16	2.83	0.33	0.20–.46	0.49	0.30–0.65	0.69	0.65–0.72
Assessment – Therapy 2 (*N* = 95)	3.04	4.55	4.41	0.67	0.48–0.86	0.69	0.49–0.90	0.75	0.71–0.78
Assessment – Therapy 3 (*N* = 66)	3.75	4.07	4.21	0.92	0.69–1.15	0.89	0.63–1.08	0.81	0.77–0.84
Assessment – Therapy 4 (*N* = 36)	4.37	4.24	4.32	1.03	0.60–1.41	1.01	0.63–1.36	0.84	0.80–0.88

Bootstrap confidence interval (1000 iterations)

Table 3 reports the results of individual level analysis, showing the proportion of participants with a large improvement by Cohen’s D effect size (ES > 0.5) and the proportion of participants with a change > 2.77 SEM using a Cronbach’s alpha score of 0.931 derived from this data, which is comparable to previously calculated reliability statistics. Both approaches indicated that the proportion of patients with a significant improvement increased with the progress of therapy, ranging from 38.1% (95% CI; 29.9–46.3) to 72.2% (95% CI; 58.3–86.1) when > 0.5 effect size was used and from 27.6% (95% CI; 20.1–35.8) to 66.7% (95% CI; 50.0–80.6) when > 2.77 SEM was used. At each time point the proportion with a large improvement by effect size was 6 to 10% higher than the proportion with a large improvement using SEM > 2.77 approach. Agreement between methods was examined using Kappa statistics; substantial agreement was shown at therapy 1 (0.795), therapy 2 (0.874) and therapy 3 (0.784); and moderate agreement at therapy 4 (0.609).

**Table 3:** Evaluation of responsiveness of SWEMWBS at individual level

Large Improvement (Cohen’s D > .5)					SEM Improved > 2.77 SEM (2.87 points)		
	N	N improved	% improved	95% CI	N improved	% improved	95% CI
Assessment – Therapy 1	134	51	38.1	29.9–46.3	37	27.6	20.1–35.8
Assessment – Therapy 2	95	52	54.7	44.2–65.3	46	48.4	38.9–58.9
Assessment – Therapy 3	66	42	63.6	51.5–75.8	35	53.0	40.9–65.2
Assessment – Therapy 4	36	26	72.2	58.3–86.1	24	66.7	50.0–80.6

Given the discrepancy between the two standards and in line with other literature [21], lower thresholds of 1 and 2 SEM were also examined (see Table 4). These showed improvement in 51.5% (95% CI; 43.0–59.8) to 72.2% (95% CI; 56.0–84.1) at a threshold of 1 SEM, and in 38.1% (95% CI; 30.2–46.5) to 77.8% (95% CI; 61.9–88.2) at a threshold of 2 SEM. at a threshold of 2 SEM.

**Table 4:** Improvement at lower SEM thresholds

SEM Improved at 1 SEM (1.03 points)					SEM Improved at 2 SEM (2.06 points)		
	N	N improved	% improved	95% CI	N improved	% improved	95% CI
Assessment – Therapy 1	134	69	51.5	43–59.8%	51	38.1	30.2–46.5%
Assessment – Therapy 2	95	66	69.5%	59.6–77.8%	56	58.9	48.9–68.3%
Assessment – Therapy 3	66	49	74.2	62.6–83.2%	42	63.6	51.5–74.1%
Assessment – Therapy 4	36	26	72.2	56–84.1%	28	77.8	61.9–88.2%

## Discussion

### Summary of key findings

English populations norms for SWEMWBS indicate mean (SD) as 23.7 (3.92) for men and 23.2 (3.99) for women [24]. Participants in this study had scores of 19.3 (3.9) at baseline rising to 23.3 (4.9), indicating low mental wellbeing before treatment and, given the predominantly female sample, population average levels after 4 sessions.

Assessment of group level responsiveness using Cohen’s D effect size indicated increasing improvement from assessment at each therapy session (ES = 0.33–1.03 and using SRM = 0.49–1.01). We evaluated the significance of the SRM using the probability of change statistic P to range between 0.65–0.8, above 0.5 at every time point, indicating the ability to detect change. This responsiveness can be compared to that of WEMWBS which Maheswaran et al. found to have a probability of change statistic above 0.7 in all studies [9, 25]. Both methods confirmed that SWEMWBS is able to detect change at group level between each therapy session and that responsiveness increased gradually with each session.

Assessment of individual level responsiveness indicated SWEMWBS ability to detect change at each time point, with improvement of 38.1–72.2% using Cohen’s D as a standard cf. 27.6–66.7% by 2.77 SEM. Use of the Cohen’s D standard overestimated the proportions in comparison to 2.77 SEM at each time point by 6 to 10%. The agreement between two methods was found to be moderate to substantial regardless of this. Further analysis using a lower threshold indicated that Cohens D underestimated ability to detect change compared to a threshold of 1 SEM threshold and approximately equated to ability to detect change at a 2 SEM threshold.

### Discussion of methods used

Cohen’s D effect size is dependent on between-subject variability, whilst the SRM is dependent on within-subject variability [26]. A limitation of using effect size as a standard is that it can be influenced by the heterogeneity of the sample, with a larger baseline standard deviation resulting in a smaller effect size. This means that the effect size standard does not account for variation in individual change scores [14]. The SRM approach takes into account the variability in individual change scores. However as a result of this, comparable individual changes have different SRM values depending upon the variability of change in the sample [14]. As SRM and SEM based methods take into account between person differences rather than between group differences, it is likely to be preferable to use SRM and SEM thresholds when looking at before and after change. We found that after each therapy session effect size and SRM produced more or less comparable values showing an increasing trend, providing corroboration of responsiveness via the two methods.

We considered change scores greater than 1 SEM [21] as well as 2.77 SEM [22] as cut off for statistically meaningful change at individual level as previous research has suggested both. The discrepancy between results might be due to differing methodologies, but could be explained by the fact that 2.77 SEM accounts for measurement error, combined variability across scores and chance at 95% CI and therefore represents a higher threshold for meaningful change than 0.5 Cohen’s D. It has also been suggested that thresholds of as low as 0.2 Cohen’s D may be sufficient to demonstrate change [19]. Given this, the lower recommended threshold of 1 SEM (a change score of 1.03 points), or 2 SEM (change score of 2.06 points) which is close to the 95% confidence interval, rather than the change score of 2.87 suggested by 2.77SEM may be taken to denote statistically meaningful change.

### Strengths and limitations

The study is based upon longitudinal data collected during practice of cognitive hypnotherapy with no control data. The changes could indicate regression to the mean or spontaneous improvement in mental health. As our objective was to determine whether SWEMWBS could detect changes in mental well-being for whatever reason, not whether this effect was due to administration of CHT, this is not of significant consequence to the analysis.

Crosby et. Al. have argued that an ideal assessment of responsiveness would involve integration of anchor based and distribution based techniques [14]. Responsiveness in this study was assessed using the distribution based methods only; and does not take into account the minimum important change as per the standards of programme participants, service users, carers or clinicians. Traditionally this would be done using a Global Rating of Change scale, although questions have been raised as to whether this method is valid for scales of mental wellbeing [27]. In the absence of an appropriate anchor, distribution methods are considered most appropriate [21, 25]. Previous literature has suggested that an improvement of 0.5 units on each item on a Likert scale would equate to an improvement deemed important by individuals [28], which would equate to 3.5 SWEMWBS points which is higher than the threshold derived from even the most stringent tests in this study. Future studies using anchor based methods are need to refine these estimates and confirm the change score indicative of minimally important change from the perspective of study participants.

Studies comparing the responsiveness of SWEMWBS using the recommended two week as opposed to a one week response option are also needed to substantiate these findings.

### Implications for practice

SWEMWBS is an attractive candidate for use as a clinical outcome measure due to its brevity and popularity with patients [6], allowing data to be easily collected in busy clinical settings. Despite the fact that SWEMWBS was originally developed to measure mental wellbeing at the population level, results indicate that it is responsive to change at both group and individual level in a clinical sample and in both group and individual analyses, responsiveness improved with prolonged therapy.

## Conclusion

SWEMWBS is responsive to change at group level and individual level in a clinical sample of patients with depression and anxiety. Results using different standards suggest a difference of either 1 or 3 points as the threshold for statistically meaningful change at the individual level.

## Abbreviations

(P^): Probability of change statistic
CHT: Cognitive Hypnotherapy Treatment
CI: Confidence interval
ES: Effect Size
QCH: Quest Cognitive Hypnotherapy
SD: Standard Deviation
SEM: Standard Error of the Mean
SRM: Standardised Response Mean
SWEMWBS: Short Warwick-Edinburgh Mental Wellbeing Scale
WEMWBS: The Warwick-Edinburgh Mental Wellbeing Scale
